# Acetonitrile­[2-(1-{bis­[2,4,6-tris­(trifluoro­meth­yl)phen­yl]phosphan­yloxy}-1-methyl­eth­yl)pyridine]­methyl­palladium(II) hexa­fluoridoanti­monate dichloro­methane hemisolvate

**DOI:** 10.1107/S1600536811005277

**Published:** 2011-02-23

**Authors:** Liuzhong Li, Peter S. White, Aiyou Hao

**Affiliations:** a School of Chemistry and Chemical Engineering, Shandong University, Jinan 250100, People’s Republic of China; b Department of Chemistry, The University of North Carolina at Chapel Hill, Chapel Hill, North Carolina 27599, USA

## Abstract

In the title compound, [Pd(CH_3_)(C_26_H_14_F_18_NOP)(C_2_H_3_N)][SbF_6_]·0.5CH_2_Cl_2_, the Pd^2+^ cation has a distorted square-planar environment, being coordinated by the acetonitrile [Pd—N = 2.078 (8) Å] and methyl [Pd—C = 2.052 (9) Å] groups and the bidentate ligand 2-(1-{bis­[2,4,6-tris­(trifluoro­meth­yl)phen­yl]phosphan­yloxy}-1-methyl­eth­yl)pyridine (*L*). In *L*, one –CF_3_ group is rotationally disordered between two orientations in a 1:1 ratio. The solvent mol­ecule was treated as disordered between two positions related by an inversion center with occupancies fixed at 0.5. The crystal packing exhibits weak inter­molecular C—H⋯F contacts.

## Related literature

For general background to the chemistry of phosphine-imine ligands and palladium complexes, see: Batsanov *et al.* (2002 ▶); Chen *et al.* (2003 ▶); Doherty *et al.* (2007 ▶); Flapper *et al.* (2009*a* ▶,*b* ▶); Guan & Marshall (2002 ▶); Kermagoret & Braunstein (2008 ▶); Speiser *et al.* (2004 ▶).
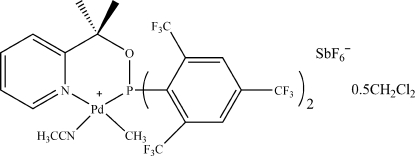


## Experimental

### 

#### Crystal data

[Pd(CH_3_)(C_26_H_14_F_18_NOP)(C_2_H_3_N)][SbF_6_]·0.5CH_2_Cl_2_
*M* *_r_* = 1170.05
Triclinic, 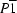

*a* = 8.8635 (8) Å
*b* = 12.1336 (12) Å
*c* = 19.107 (2) Å
α = 79.166 (8)°
β = 80.147 (8)°
γ = 78.266 (8)°
*V* = 1957.2 (3) Å^3^
*Z* = 2
Cu *K*α radiation
μ = 11.56 mm^−1^
*T* = 100 K
0.15 × 0.10 × 0.05 mm

#### Data collection

Bruker APEXII CCD diffractometer
Absorption correction: numerical (*SADABS*; Bruker, 2007 ▶) *T*_min_ = 0.276, *T*_max_ = 0.596
12653 measured reflections
6232 independent reflections
4802 reflections with *I* > 2σ(*I*)
*R*_int_ = 0.052

#### Refinement

*R*[*F* ^2^ > 2σ(*F* ^2^)] = 0.066
*wR*(*F* ^2^) = 0.191
*S* = 1.02
6232 reflections
590 parameters
63 restraints
H-atom parameters constrained
Δρ_max_ = 2.34 e Å^−3^
Δρ_min_ = −0.95 e Å^−3^

### 

Data collection: *APEX2* (Bruker, 2007 ▶); cell refinement: *SAINT* (Bruker, 2007 ▶); data reduction: *SAINT*; program(s) used to solve structure: *SHELXS97* (Sheldrick, 2008 ▶); program(s) used to refine structure: *SHELXL97* (Sheldrick, 2008 ▶); molecular graphics: *SHELXTL* (Sheldrick, 2008 ▶); software used to prepare material for publication: *SHELXTL*.

## Supplementary Material

Crystal structure: contains datablocks I, global. DOI: 10.1107/S1600536811005277/cv5026sup1.cif

Structure factors: contains datablocks I. DOI: 10.1107/S1600536811005277/cv5026Isup2.hkl

Additional supplementary materials:  crystallographic information; 3D view; checkCIF report

## Figures and Tables

**Table 1:** Hydrogen-bond geometry (Å, °)

*D*—H⋯*A*	*D*—H	H⋯*A*	*D*⋯*A*	*D*—H⋯*A*
C4—H4*B*⋯F1^i^	0.98	2.40	3.365 (13)	166
C12—H12⋯F5^i^	0.95	2.60	3.276 (11)	129
C4—H4*A*⋯F4	0.98	2.53	3.464 (13)	158
C4—H4*C*⋯F2^ii^	0.98	2.30	3.225 (13)	155
C11—H11*C*⋯F5^iii^	0.98	2.49	3.352 (11)	146

Symmetry codes: (i) 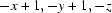; (ii) 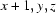; (iii) 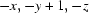.

**Fig. 1.**
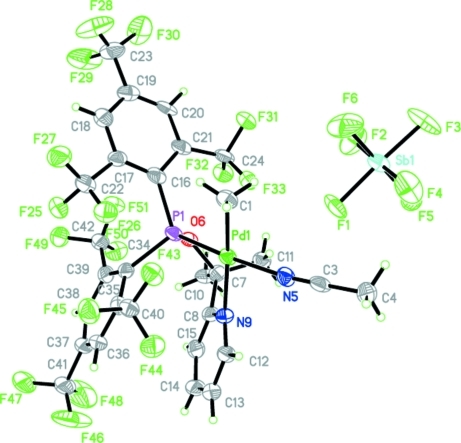
The molecular structure of (I) showing the atom-numbering scheme and 50% probabilty displacement ellipsoids. Only one part of the disordered CF3 group is shown. The H atoms and solvent molecules were omitted for clarity.

## supplementary crystallographic information

### Comment

The oligomerization of ethylene is one of the most important industrial processes to obtain linear alpha-olefins (Chen *et al.*, 2003; Guan *et al.*,2002). Bidentate phosphine-imine ligands (P^N ligands) have attracted considerable concerns in the field of transition metal catalysis. Palladium and nickel complexes with P^N ligands have been widely applied in the oligomerization and polymerization of ethylene(Doherty *et al.*, 2007; Flapper *et al.*, 2009*a*,*b*; Kermagoret *et al.*, 2008; Speiser *et al.*, 2004). Herein, we report the synthesis and characterization of cationic palladium complex, with electron-poor bulky ligand bearing tris(trifluoromethyl) phenyl phosphine, which can be synthesized according to the literature (Batsanov *et al.*, 2002; Speiser *et al.*, 2004), [(CH_3_N)(CH_3_)*L*Pd]^+^ [SbF_6_]^-^ 0.5(CH_2_Cl_2_), where *L* = [2-ethyl-(1'-methyl-1'-oxy(bis(2,4,6-tris(trifluoromethyl) phenyl)phosphino)]pyridine.

In the title compound, each Pd center has a distorted square-planar environment being coordinated by acetonitrile [Pd—N 2.078 (8) Å], methyl [Pd—C 2.052 (9) Å] and bidentate ligand *L*. In *L*, one CF_3_ group is rotationally disordered between two orientations in a ratio 1:1. The solvent molecule has been treated as disordered between two positions related by inversion center with occupancies fixed to 0.5 each. The crystal packing exhibits weak intermolecular C—H···F contacts (Table 1).

### Experimental

All manipulations of air- and/or moisture-sensitive compounds were conducted using standard Schlenk techniques. Argon was purified by passage through columns of BASF R3–11 catalyst (Chemalog) and 4Å molecular sieves. All solvents were deoxygenated, dried and distilled using common techniques. 2-Pyridin-2-ylpropan-2-ol and di[tris(trifluoromethyl)phenyl]phosphine chloride were prepared according to the literature procedures(Batsanov *et al.*, 2002; Speiser *et al.*, 2004). A flame-dried Schlenk flask was charged with 2-pyridin-2-ylpropan-2-ol (280 mg, 1.30 mmol) and dried THF (5 ml). The solution was cooled to -78°C, and 2.5 mol/l n-BuLi in hexane (0.52 ml, 1.30 mmol) was added slowly. After stirring of 2.0 hrs at -78°C, 800 mg in THF(2 ml) was added slowly. Stirring for 1 day, 30 ml degassed saturated NaCl solution was charged for hydrolysis. After separation, dry and column purification, the ligand of 2-ethyl-[1'-methyl-1'-oxy(di(2, 4, 6-tris(trifluoromethyl) phenyl)phosphino)] pyridine was obtained. The cationic complex was prepared by reaction of the above ligand (1.0 equiv.), (COD)PdMeCl (1.0 equiv.), and AgSbF_6_ (1.0 equiv.) at RT, and the single-crystal was cultivated by recrystallization of CH_2_Cl_2_ and pentane. Calcd for C29H20F24N2OPPdSb: C, 30.89; H, 1.79; N, 2.48. Found: C, 30.89; H, 1.59; N, 2.21.

### Refinement

All H atoms were geometrically positioned (C—H 0.95-0.99 Å) and refined as riding, with Uiso(H) = 1.2-1.5 Ueq(C).

### Crystal data

**Table:**

[Pd(CH_3_)(C_26_H_14_F_18_NOP)(C_2_H_3_N)][SbF_6_]·0.5CH_2_Cl_2_	*Z* = 2
*M**_r_* = 1170.05	*F*(000) = 1130
Triclinic, *P*1	*D*_x_ = 1.985 Mg m^−^^3^
Hall symbol: -P 1	Cu *K*α radiation, λ = 1.54178 Å
*a* = 8.8635 (8) Å	Cell parameters from 3085 reflections
*b* = 12.1336 (12) Å	θ = 2.4–66.3°
*c* = 19.107 (2) Å	µ = 11.56 mm^−^^1^
α = 79.166 (8)°	*T* = 100 K
β = 80.147 (8)°	Prism, colourless
γ = 78.266 (8)°	0.15 × 0.10 × 0.05 mm
*V* = 1957.2 (3) Å^3^	

### Data collection

**Table:**

Bruker APEXII CCD diffractometer	6232 independent reflections
Radiation source: fine-focus sealed tube	4802 reflections with *I* > 2σ(*I*)
graphite	*R*_int_ = 0.052
φ and ω scans	θ_max_ = 67.3°, θ_min_ = 2.4°
Absorption correction: numerical (*SADABS*; Bruker, 2007)	*h* = −10→10
*T*_min_ = 0.276, *T*_max_ = 0.596	*k* = −14→13
12653 measured reflections	*l* = −22→22

### Refinement

**Table:**

Refinement on *F*^2^	Primary atom site location: structure-invariant direct methods
Least-squares matrix: full	Secondary atom site location: difference Fourier map
*R*[*F*^2^ > 2σ(*F*^2^)] = 0.066	Hydrogen site location: inferred from neighbouring sites
*wR*(*F*^2^) = 0.191	H-atom parameters constrained
*S* = 1.02	*w* = 1/[σ^2^(*F*_o_^2^) + (0.1096*P*)^2^ + 11.4198*P*] where *P* = (*F*_o_^2^ + 2*F*_c_^2^)/3
6232 reflections	(Δ/σ)_max_ < 0.001
590 parameters	Δρ_max_ = 2.34 e Å^−^^3^
63 restraints	Δρ_min_ = −0.95 e Å^−^^3^

### Special details

**Table:**

Geometry. All e.s.d.'s (except the e.s.d. in the dihedral angle between two l.s. planes) are estimated using the full covariance matrix. The cell e.s.d.'s are taken into account individually in the estimation of e.s.d.'s in distances, angles and torsion angles; correlations between e.s.d.'s in cell parameters are only used when they are defined by crystal symmetry. An approximate (isotropic) treatment of cell e.s.d.'s is used for estimating e.s.d.'s involving l.s. planes.
Refinement. Refinement of *F*^2^ against ALL reflections. The weighted *R*-factor *wR* and goodness of fit *S* are based on *F*^2^, conventional *R*-factors *R* are based on *F*, with *F* set to zero for negative *F*^2^. The threshold expression of *F*^2^ > σ(*F*^2^) is used only for calculating *R*-factors(gt) *etc*. and is not relevant to the choice of reflections for refinement. *R*-factors based on *F*^2^ are statistically about twice as large as those based on *F*, and *R*- factors based on ALL data will be even larger.

### Fractional atomic coordinates and isotropic or equivalent isotropic displacement parameters (Å^2^)

**Table:**

	*x*	*y*	*z*	*U*_iso_*/*U*_eq_	Occ. (<1)
Pd1	0.39331 (7)	0.28306 (5)	0.20297 (4)	0.0287 (2)	
P1	0.2317 (2)	0.17257 (18)	0.26378 (12)	0.0271 (5)	
C1	0.3238 (12)	0.3978 (8)	0.2736 (5)	0.038 (2)	
H1A	0.4154	0.4136	0.2896	0.058*	
H1B	0.2561	0.3658	0.3153	0.058*	
H1C	0.2668	0.4687	0.2496	0.058*	
C3	0.5856 (11)	0.4644 (8)	0.1041 (6)	0.035 (2)	
C4	0.6566 (13)	0.5542 (9)	0.0576 (6)	0.044 (2)	
H4A	0.5830	0.6265	0.0569	0.066*	
H4B	0.6842	0.5357	0.0087	0.066*	
H4C	0.7507	0.5612	0.0757	0.066*	
N5	0.5278 (9)	0.3961 (7)	0.1395 (4)	0.0329 (17)	
O6	0.1387 (7)	0.1455 (5)	0.2067 (3)	0.0279 (13)	
C7	0.2062 (10)	0.1351 (8)	0.1295 (5)	0.0293 (19)	
C8	0.3807 (10)	0.0855 (8)	0.1224 (5)	0.0290 (19)	
N9	0.4740 (9)	0.1537 (7)	0.1327 (4)	0.0301 (16)	
C10	0.1082 (11)	0.0593 (9)	0.1097 (5)	0.035 (2)	
H10A	−0.0023	0.0895	0.1227	0.052*	
H10B	0.1336	−0.0182	0.1358	0.052*	
H10C	0.1302	0.0578	0.0578	0.052*	
C11	0.1809 (11)	0.2512 (8)	0.0820 (5)	0.034 (2)	
H11A	0.2297	0.2440	0.0326	0.052*	
H11B	0.2277	0.3047	0.1003	0.052*	
H11C	0.0691	0.2796	0.0825	0.052*	
C12	0.6264 (10)	0.1235 (8)	0.1163 (5)	0.0307 (19)	
H12	0.6913	0.1744	0.1209	0.037*	
C13	0.6963 (11)	0.0210 (9)	0.0926 (5)	0.037 (2)	
H13	0.8062	0.0018	0.0815	0.045*	
C14	0.6010 (12)	−0.0513 (9)	0.0857 (5)	0.038 (2)	
H14	0.6443	−0.1237	0.0719	0.046*	
C15	0.4404 (12)	−0.0178 (8)	0.0991 (5)	0.036 (2)	
H15	0.3727	−0.0652	0.0923	0.044*	
C16	0.0614 (11)	0.2221 (7)	0.3317 (5)	0.032 (2)	
C17	0.0542 (11)	0.1885 (8)	0.4067 (5)	0.032 (2)	
C18	−0.0813 (12)	0.2128 (8)	0.4534 (6)	0.038 (2)	
H18	−0.0823	0.1875	0.5037	0.045*	
C19	−0.2166 (12)	0.2743 (9)	0.4271 (6)	0.040 (2)	
C20	−0.2084 (11)	0.3147 (8)	0.3550 (6)	0.036 (2)	
H20	−0.2984	0.3604	0.3372	0.043*	
C21	−0.0762 (11)	0.2923 (8)	0.3076 (6)	0.034 (2)	
C22	0.1891 (12)	0.1192 (9)	0.4428 (6)	0.039 (2)	
C23	−0.3671 (13)	0.2958 (11)	0.4782 (6)	0.049 (3)	
C24	−0.0904 (11)	0.3491 (8)	0.2309 (6)	0.037 (2)	
F25	0.1962 (7)	0.0064 (5)	0.4459 (3)	0.0479 (15)	
F26	0.3246 (6)	0.1442 (5)	0.4090 (3)	0.0425 (13)	
F27	0.1776 (8)	0.1381 (6)	0.5101 (3)	0.0548 (17)	
F28	−0.346 (2)	0.323 (2)	0.5373 (11)	0.081 (5)	0.50
F29	−0.4321 (18)	0.1986 (15)	0.4936 (10)	0.068 (4)	0.50
F30	−0.4754 (19)	0.3697 (17)	0.4483 (9)	0.070 (4)	0.50
F28A	−0.354 (2)	0.2539 (18)	0.5459 (9)	0.072 (5)	0.50
F29A	−0.476 (3)	0.263 (3)	0.4619 (14)	0.103 (7)	0.50
F30A	−0.411 (2)	0.4029 (17)	0.4800 (12)	0.091 (6)	0.50
F31	−0.1953 (7)	0.4478 (5)	0.2306 (3)	0.0470 (15)	
F32	−0.1387 (7)	0.2859 (5)	0.1925 (3)	0.0404 (13)	
F33	0.0422 (6)	0.3815 (5)	0.1958 (3)	0.0382 (12)	
C34	0.3269 (10)	0.0207 (8)	0.2948 (5)	0.0274 (18)	
C35	0.4889 (11)	−0.0100 (8)	0.2957 (5)	0.033 (2)	
C36	0.5675 (12)	−0.1170 (9)	0.2856 (5)	0.041 (2)	
H36	0.6771	−0.1342	0.2851	0.049*	
C37	0.4903 (13)	−0.1995 (8)	0.2763 (6)	0.043 (3)	
C38	0.3305 (13)	−0.1775 (9)	0.2834 (6)	0.041 (2)	
H38	0.2757	−0.2360	0.2811	0.049*	
C39	0.2482 (12)	−0.0697 (8)	0.2941 (5)	0.035 (2)	
C40	0.5991 (11)	0.0602 (9)	0.3100 (6)	0.039 (2)	
C41	0.5765 (16)	−0.3156 (10)	0.2601 (8)	0.060 (4)	
C42	0.0767 (12)	−0.0694 (8)	0.3098 (5)	0.037 (2)	
F43	0.5466 (7)	0.1693 (5)	0.3180 (3)	0.0430 (13)	
F44	0.7231 (7)	0.0620 (6)	0.2587 (3)	0.0486 (15)	
F45	0.6537 (7)	0.0103 (6)	0.3723 (3)	0.0490 (15)	
F46	0.7237 (11)	−0.3168 (7)	0.2408 (8)	0.121 (5)	
F47	0.5548 (11)	−0.3967 (6)	0.3154 (5)	0.080 (3)	
F48	0.5253 (16)	−0.3471 (8)	0.2071 (6)	0.114 (4)	
F49	0.0470 (7)	−0.1512 (5)	0.3669 (3)	0.0488 (15)	
F50	0.0170 (7)	−0.0935 (5)	0.2562 (3)	0.0456 (14)	
F51	−0.0107 (6)	0.0275 (5)	0.3278 (3)	0.0381 (13)	
Sb1	0.13293 (7)	0.70526 (5)	0.07820 (4)	0.0384 (2)	
F1	0.2407 (9)	0.5556 (5)	0.0969 (4)	0.0581 (18)	
F2	−0.0566 (8)	0.6539 (8)	0.0901 (4)	0.068 (2)	
F3	0.0296 (10)	0.8560 (6)	0.0551 (5)	0.078 (3)	
F4	0.3226 (8)	0.7596 (7)	0.0633 (5)	0.071 (2)	
F5	0.1630 (7)	0.6941 (6)	−0.0202 (3)	0.0465 (14)	
F6	0.1099 (11)	0.7165 (9)	0.1756 (4)	0.085 (3)	
C52	0.026 (3)	0.411 (3)	0.6363 (14)	0.077 (8)	0.50
H52A	0.0107	0.3478	0.6766	0.093*	0.50
H52B	0.0313	0.4793	0.6562	0.093*	0.50
Cl1	−0.1188 (19)	0.4375 (15)	0.5867 (9)	0.169 (6)	0.50
Cl2	0.190 (2)	0.374 (3)	0.5796 (15)	0.273 (13)	0.50

### Atomic displacement parameters (Å^2^)

**Table:**

	*U*^11^	*U*^22^	*U*^33^	*U*^12^	*U*^13^	*U*^23^
Pd1	0.0282 (3)	0.0193 (3)	0.0379 (4)	−0.0050 (3)	−0.0009 (3)	−0.0052 (3)
P1	0.0243 (10)	0.0179 (10)	0.0382 (12)	−0.0025 (9)	−0.0017 (9)	−0.0058 (9)
C1	0.048 (6)	0.025 (5)	0.043 (5)	−0.006 (4)	−0.002 (4)	−0.014 (4)
C3	0.031 (5)	0.023 (5)	0.050 (6)	0.001 (4)	−0.001 (4)	−0.014 (4)
C4	0.050 (6)	0.025 (5)	0.054 (6)	−0.013 (5)	0.008 (5)	−0.006 (4)
N5	0.029 (4)	0.023 (4)	0.046 (4)	−0.008 (4)	−0.003 (3)	−0.002 (3)
O6	0.023 (3)	0.023 (3)	0.038 (3)	−0.002 (2)	−0.001 (2)	−0.008 (2)
C7	0.030 (4)	0.033 (5)	0.027 (4)	−0.007 (4)	−0.004 (3)	−0.007 (4)
C8	0.029 (4)	0.028 (5)	0.030 (4)	−0.004 (4)	0.000 (3)	−0.008 (4)
N9	0.032 (4)	0.029 (4)	0.027 (4)	−0.001 (3)	0.000 (3)	−0.009 (3)
C10	0.028 (4)	0.038 (5)	0.043 (5)	−0.006 (4)	−0.001 (4)	−0.020 (4)
C11	0.031 (5)	0.034 (5)	0.037 (5)	−0.002 (4)	−0.007 (4)	−0.005 (4)
C12	0.022 (4)	0.025 (5)	0.044 (5)	−0.008 (4)	0.000 (4)	−0.004 (4)
C13	0.033 (5)	0.035 (5)	0.039 (5)	−0.001 (4)	0.002 (4)	−0.004 (4)
C14	0.043 (5)	0.028 (5)	0.042 (5)	−0.002 (4)	0.003 (4)	−0.015 (4)
C15	0.041 (5)	0.027 (5)	0.040 (5)	−0.009 (4)	0.000 (4)	−0.005 (4)
C16	0.034 (5)	0.016 (4)	0.048 (5)	−0.006 (4)	−0.004 (4)	−0.009 (4)
C17	0.033 (5)	0.024 (4)	0.038 (5)	−0.004 (4)	0.002 (4)	−0.010 (4)
C18	0.041 (5)	0.029 (5)	0.042 (5)	−0.005 (4)	−0.005 (4)	−0.007 (4)
C19	0.034 (5)	0.036 (6)	0.052 (6)	−0.003 (4)	0.002 (4)	−0.019 (5)
C20	0.027 (5)	0.021 (5)	0.056 (6)	0.009 (4)	−0.006 (4)	−0.013 (4)
C21	0.030 (5)	0.023 (5)	0.049 (6)	0.001 (4)	−0.002 (4)	−0.013 (4)
C22	0.036 (5)	0.033 (5)	0.044 (6)	0.002 (4)	−0.005 (4)	−0.007 (4)
C23	0.038 (5)	0.059 (6)	0.047 (5)	0.002 (5)	0.001 (4)	−0.019 (5)
C24	0.030 (5)	0.026 (5)	0.050 (6)	0.007 (4)	−0.002 (4)	−0.010 (4)
F25	0.051 (3)	0.035 (3)	0.049 (3)	0.001 (3)	−0.003 (3)	0.003 (3)
F26	0.034 (3)	0.047 (4)	0.048 (3)	−0.005 (3)	−0.009 (2)	−0.010 (3)
F27	0.054 (4)	0.067 (5)	0.039 (3)	0.008 (3)	−0.010 (3)	−0.014 (3)
F28	0.078 (9)	0.093 (9)	0.070 (8)	−0.011 (8)	0.012 (7)	−0.037 (8)
F29	0.048 (7)	0.059 (7)	0.083 (8)	−0.010 (6)	0.021 (6)	−0.008 (6)
F30	0.048 (7)	0.074 (8)	0.067 (7)	0.014 (6)	0.008 (6)	−0.001 (6)
F28A	0.062 (7)	0.075 (8)	0.055 (7)	0.009 (7)	0.017 (6)	0.004 (7)
F29A	0.088 (10)	0.125 (11)	0.106 (10)	−0.030 (9)	−0.002 (8)	−0.041 (9)
F30A	0.075 (8)	0.074 (9)	0.097 (9)	0.018 (7)	0.031 (7)	−0.021 (7)
F31	0.044 (3)	0.033 (3)	0.053 (4)	0.012 (3)	−0.004 (3)	−0.004 (3)
F32	0.039 (3)	0.038 (3)	0.045 (3)	−0.003 (3)	−0.009 (2)	−0.010 (3)
F33	0.038 (3)	0.028 (3)	0.044 (3)	−0.001 (2)	−0.004 (2)	0.000 (2)
C34	0.029 (4)	0.023 (4)	0.029 (4)	−0.006 (4)	0.003 (3)	−0.005 (3)
C35	0.034 (5)	0.030 (5)	0.033 (5)	−0.006 (4)	−0.005 (4)	0.003 (4)
C36	0.033 (5)	0.034 (6)	0.042 (5)	0.009 (4)	0.006 (4)	0.002 (4)
C37	0.050 (6)	0.021 (5)	0.046 (6)	0.004 (5)	0.012 (5)	−0.004 (4)
C38	0.050 (6)	0.026 (5)	0.043 (6)	−0.011 (5)	0.004 (4)	−0.005 (4)
C39	0.046 (6)	0.023 (5)	0.034 (5)	−0.007 (4)	0.004 (4)	−0.003 (4)
C40	0.027 (5)	0.038 (6)	0.045 (5)	0.004 (4)	−0.005 (4)	0.000 (4)
C41	0.063 (8)	0.025 (6)	0.074 (9)	0.006 (6)	0.020 (6)	−0.003 (5)
C42	0.044 (5)	0.025 (5)	0.041 (5)	−0.003 (4)	−0.001 (4)	−0.008 (4)
F43	0.041 (3)	0.036 (3)	0.054 (3)	−0.011 (3)	−0.009 (3)	−0.004 (3)
F44	0.032 (3)	0.058 (4)	0.054 (4)	−0.011 (3)	−0.002 (3)	−0.006 (3)
F45	0.046 (3)	0.048 (4)	0.050 (4)	−0.006 (3)	−0.014 (3)	0.003 (3)
F46	0.061 (5)	0.036 (4)	0.237 (14)	−0.002 (4)	0.065 (7)	−0.033 (6)
F47	0.093 (6)	0.024 (3)	0.096 (6)	0.009 (4)	0.023 (5)	0.003 (3)
F48	0.167 (11)	0.063 (6)	0.106 (7)	0.033 (7)	−0.023 (7)	−0.052 (6)
F49	0.046 (3)	0.039 (3)	0.054 (4)	−0.011 (3)	0.009 (3)	0.002 (3)
F50	0.045 (3)	0.043 (3)	0.054 (4)	−0.018 (3)	0.002 (3)	−0.019 (3)
F51	0.025 (3)	0.028 (3)	0.061 (4)	−0.005 (2)	0.002 (2)	−0.013 (3)
Sb1	0.0345 (4)	0.0292 (4)	0.0533 (4)	0.0007 (3)	−0.0098 (3)	−0.0143 (3)
F1	0.076 (5)	0.024 (3)	0.068 (4)	0.013 (3)	−0.024 (4)	−0.003 (3)
F2	0.040 (3)	0.106 (7)	0.063 (4)	−0.035 (4)	−0.001 (3)	−0.008 (4)
F3	0.079 (5)	0.037 (4)	0.123 (7)	0.027 (4)	−0.048 (5)	−0.033 (4)
F4	0.048 (4)	0.055 (4)	0.120 (7)	−0.021 (4)	−0.034 (4)	−0.002 (4)
F5	0.044 (3)	0.049 (4)	0.044 (3)	−0.006 (3)	−0.004 (3)	−0.004 (3)
F6	0.088 (6)	0.124 (8)	0.046 (4)	0.011 (5)	−0.014 (4)	−0.045 (5)
C52	0.082 (12)	0.069 (11)	0.090 (12)	−0.023 (9)	−0.008 (9)	−0.030 (9)
Cl1	0.186 (10)	0.154 (9)	0.173 (9)	−0.001 (8)	−0.025 (8)	−0.071 (8)
Cl2	0.272 (15)	0.281 (16)	0.277 (15)	−0.060 (10)	−0.038 (10)	−0.059 (10)

### Geometric parameters (Å, °)

**Table:**

Pd1—C1	2.052 (9)	C22—F27	1.332 (12)
Pd1—N5	2.078 (8)	C22—F25	1.348 (12)
Pd1—N9	2.187 (7)	C23—F29A	1.23 (3)
Pd1—P1	2.197 (2)	C23—F30A	1.28 (2)
P1—O6	1.591 (6)	C23—F28	1.29 (2)
P1—C16	1.891 (9)	C23—F30	1.30 (2)
P1—C34	1.894 (9)	C23—F28A	1.31 (2)
C1—H1A	0.9800	C23—F29	1.38 (2)
C1—H1B	0.9800	C24—F32	1.328 (12)
C1—H1C	0.9800	C24—F33	1.343 (11)
C3—N5	1.118 (13)	C24—F31	1.358 (11)
C3—C4	1.448 (14)	F28—F28A	0.84 (2)
C4—H4A	0.9800	F28—F30A	1.43 (3)
C4—H4B	0.9800	F29—F29A	0.95 (3)
C4—H4C	0.9800	F29—F28A	1.61 (3)
O6—C7	1.513 (10)	F30—F30A	1.08 (3)
C7—C10	1.519 (12)	F30—F29A	1.28 (3)
C7—C11	1.523 (13)	C34—C35	1.410 (13)
C7—C8	1.533 (12)	C34—C39	1.418 (14)
C8—N9	1.343 (12)	C35—C36	1.374 (14)
C8—C15	1.381 (13)	C35—C40	1.507 (15)
N9—C12	1.324 (12)	C36—C37	1.375 (16)
C10—H10A	0.9800	C36—H36	0.9500
C10—H10B	0.9800	C37—C38	1.375 (16)
C10—H10C	0.9800	C37—C41	1.520 (14)
C11—H11A	0.9800	C38—C39	1.394 (14)
C11—H11B	0.9800	C38—H38	0.9500
C11—H11C	0.9800	C39—C42	1.498 (14)
C12—C13	1.392 (14)	C40—F43	1.340 (12)
C12—H12	0.9500	C40—F44	1.342 (12)
C13—C14	1.373 (15)	C40—F45	1.351 (12)
C13—H13	0.9500	C41—F46	1.292 (16)
C14—C15	1.392 (14)	C41—F47	1.318 (14)
C14—H14	0.9500	C41—F48	1.322 (19)
C15—H15	0.9500	C42—F50	1.332 (12)
C16—C17	1.409 (14)	C42—F51	1.338 (11)
C16—C21	1.430 (14)	C42—F49	1.359 (12)
C17—C18	1.384 (14)	Sb1—F6	1.865 (7)
C17—C22	1.507 (14)	Sb1—F2	1.872 (7)
C18—C19	1.396 (15)	Sb1—F1	1.873 (6)
C18—H18	0.9500	Sb1—F3	1.880 (7)
C19—C20	1.367 (16)	Sb1—F5	1.880 (6)
C19—C23	1.519 (14)	Sb1—F4	1.887 (7)
C20—C21	1.367 (14)	C52—Cl1	1.666 (18)
C20—H20	0.9500	C52—Cl2	1.692 (18)
C21—C24	1.512 (15)	C52—H52A	0.9900
C22—F26	1.327 (12)	C52—H52B	0.9900
			
C1—Pd1—N5	88.1 (4)	F28—C23—F28A	37.6 (12)
C1—Pd1—N9	176.6 (4)	F30—C23—F28A	131.5 (13)
N5—Pd1—N9	93.3 (3)	F29A—C23—F29	42.4 (15)
C1—Pd1—P1	91.8 (3)	F30A—C23—F29	138.1 (15)
N5—Pd1—P1	174.1 (2)	F28—C23—F29	109.0 (16)
N9—Pd1—P1	87.0 (2)	F30—C23—F29	101.6 (15)
O6—P1—C16	99.1 (4)	F28A—C23—F29	73.5 (15)
O6—P1—C34	97.4 (4)	F29A—C23—C19	114.1 (14)
C16—P1—C34	111.1 (4)	F30A—C23—C19	110.6 (13)
O6—P1—Pd1	106.3 (3)	F28—C23—C19	112.6 (13)
C16—P1—Pd1	123.4 (3)	F30—C23—C19	112.9 (12)
C34—P1—Pd1	114.5 (3)	F28A—C23—C19	114.2 (12)
Pd1—C1—H1A	109.5	F29—C23—C19	108.7 (11)
Pd1—C1—H1B	109.5	F32—C24—F33	109.1 (8)
H1A—C1—H1B	109.5	F32—C24—F31	106.8 (8)
Pd1—C1—H1C	109.5	F33—C24—F31	104.3 (8)
H1A—C1—H1C	109.5	F32—C24—C21	113.5 (9)
H1B—C1—H1C	109.5	F33—C24—C21	112.8 (8)
N5—C3—C4	178.6 (11)	F31—C24—C21	109.8 (8)
C3—C4—H4A	109.5	F28A—F28—C23	73 (2)
C3—C4—H4B	109.5	F28A—F28—F30A	124 (3)
H4A—C4—H4B	109.5	C23—F28—F30A	55.9 (14)
C3—C4—H4C	109.5	F29A—F29—C23	60.4 (19)
H4A—C4—H4C	109.5	F29A—F29—F28A	104 (2)
H4B—C4—H4C	109.5	C23—F29—F28A	51.4 (10)
C3—N5—Pd1	172.5 (8)	F30A—F30—F29A	117 (2)
C7—O6—P1	124.2 (5)	F30A—F30—C23	64.4 (15)
O6—C7—C10	102.8 (7)	F29A—F30—C23	56.8 (14)
O6—C7—C11	110.2 (7)	F28—F28A—C23	70 (2)
C10—C7—C11	109.1 (8)	F28—F28A—F29	122 (3)
O6—C7—C8	111.6 (7)	C23—F28A—F29	55.1 (12)
C10—C7—C8	113.7 (8)	F29—F29A—C23	77 (2)
C11—C7—C8	109.2 (8)	F29—F29A—F30	137 (3)
N9—C8—C15	121.5 (9)	C23—F29A—F30	62.7 (16)
N9—C8—C7	116.1 (8)	F30—F30A—C23	66.3 (17)
C15—C8—C7	122.1 (9)	F30—F30A—F28	116 (2)
C12—N9—C8	118.8 (8)	C23—F30A—F28	56.2 (12)
C12—N9—Pd1	117.0 (6)	C35—C34—C39	115.7 (9)
C8—N9—Pd1	121.8 (6)	C35—C34—P1	121.8 (7)
C7—C10—H10A	109.5	C39—C34—P1	119.1 (7)
C7—C10—H10B	109.5	C36—C35—C34	121.6 (10)
H10A—C10—H10B	109.5	C36—C35—C40	110.1 (9)
C7—C10—H10C	109.5	C34—C35—C40	128.3 (9)
H10A—C10—H10C	109.5	C35—C36—C37	121.1 (10)
H10B—C10—H10C	109.5	C35—C36—H36	119.4
C7—C11—H11A	109.5	C37—C36—H36	119.4
C7—C11—H11B	109.5	C38—C37—C36	119.2 (9)
H11A—C11—H11B	109.5	C38—C37—C41	119.0 (11)
C7—C11—H11C	109.5	C36—C37—C41	121.8 (11)
H11A—C11—H11C	109.5	C37—C38—C39	120.4 (10)
H11B—C11—H11C	109.5	C37—C38—H38	119.8
N9—C12—C13	123.4 (9)	C39—C38—H38	119.8
N9—C12—H12	118.3	C38—C39—C34	121.1 (10)
C13—C12—H12	118.3	C38—C39—C42	111.1 (9)
C14—C13—C12	117.7 (9)	C34—C39—C42	127.6 (8)
C14—C13—H13	121.2	F43—C40—F44	105.3 (9)
C12—C13—H13	121.2	F43—C40—F45	104.7 (8)
C13—C14—C15	119.4 (9)	F44—C40—F45	106.7 (7)
C13—C14—H14	120.3	F43—C40—C35	119.5 (8)
C15—C14—H14	120.3	F44—C40—C35	111.8 (9)
C8—C15—C14	119.1 (10)	F45—C40—C35	108.0 (8)
C8—C15—H15	120.5	F46—C41—F47	109.7 (12)
C14—C15—H15	120.5	F46—C41—F48	106.4 (12)
C17—C16—C21	115.5 (9)	F47—C41—F48	104.6 (13)
C17—C16—P1	124.5 (7)	F46—C41—C37	112.0 (12)
C21—C16—P1	119.9 (7)	F47—C41—C37	111.6 (10)
C18—C17—C16	122.2 (9)	F48—C41—C37	112.2 (12)
C18—C17—C22	114.2 (9)	F50—C42—F51	106.5 (8)
C16—C17—C22	123.5 (8)	F50—C42—F49	105.6 (8)
C17—C18—C19	120.3 (10)	F51—C42—F49	105.2 (8)
C17—C18—H18	119.9	F50—C42—C39	113.9 (8)
C19—C18—H18	119.9	F51—C42—C39	115.3 (8)
C20—C19—C18	118.1 (9)	F49—C42—C39	109.4 (8)
C20—C19—C23	121.7 (10)	F6—Sb1—F2	91.6 (4)
C18—C19—C23	120.1 (10)	F6—Sb1—F1	90.2 (4)
C21—C20—C19	122.8 (9)	F2—Sb1—F1	91.3 (4)
C21—C20—H20	118.6	F6—Sb1—F3	92.2 (4)
C19—C20—H20	118.6	F2—Sb1—F3	89.8 (4)
C20—C21—C16	120.7 (10)	F1—Sb1—F3	177.4 (4)
C20—C21—C24	114.2 (9)	F6—Sb1—F5	178.2 (3)
C16—C21—C24	125.1 (9)	F2—Sb1—F5	90.2 (3)
F26—C22—F27	106.9 (8)	F1—Sb1—F5	89.2 (3)
F26—C22—F25	107.4 (8)	F3—Sb1—F5	88.4 (3)
F27—C22—F25	107.1 (8)	F6—Sb1—F4	89.9 (4)
F26—C22—C17	112.2 (8)	F2—Sb1—F4	178.2 (4)
F27—C22—C17	111.1 (8)	F1—Sb1—F4	89.8 (3)
F25—C22—C17	111.9 (9)	F3—Sb1—F4	89.0 (4)
F29A—C23—F30A	106 (2)	F5—Sb1—F4	88.3 (3)
F29A—C23—F28	131.4 (17)	Cl1—C52—Cl2	105.5 (14)
F30A—C23—F28	67.9 (16)	Cl1—C52—H52A	110.6
F29A—C23—F30	60.5 (16)	Cl2—C52—H52A	110.6
F30A—C23—F30	49.3 (13)	Cl1—C52—H52B	110.6
F28—C23—F30	111.3 (16)	Cl2—C52—H52B	110.6
F29A—C23—F28A	108.2 (19)	H52A—C52—H52B	108.8
F30A—C23—F28A	102.4 (16)		
			
C1—Pd1—P1—O6	130.4 (4)	F29A—C23—F29—F28A	−144 (2)
N5—Pd1—P1—O6	41 (2)	F30A—C23—F29—F28A	−90 (3)
N9—Pd1—P1—O6	−52.8 (3)	F28—C23—F29—F28A	−12.6 (14)
C1—Pd1—P1—C16	17.5 (5)	F30—C23—F29—F28A	−130.1 (14)
N5—Pd1—P1—C16	−72 (2)	C19—C23—F29—F28A	110.5 (13)
N9—Pd1—P1—C16	−165.7 (4)	F29A—C23—F30—F30A	156 (2)
C1—Pd1—P1—C34	−123.3 (4)	F28—C23—F30—F30A	29.8 (19)
N5—Pd1—P1—C34	147 (2)	F28A—C23—F30—F30A	67 (3)
N9—Pd1—P1—C34	53.5 (4)	F29—C23—F30—F30A	145.7 (17)
C4—C3—N5—Pd1	−12 (50)	C19—C23—F30—F30A	−98.0 (17)
C1—Pd1—N5—C3	−68 (6)	F30A—C23—F30—F29A	−156 (2)
N9—Pd1—N5—C3	115 (6)	F28—C23—F30—F29A	−126 (2)
P1—Pd1—N5—C3	22 (8)	F28A—C23—F30—F29A	−89 (3)
C16—P1—O6—C7	161.2 (6)	F29—C23—F30—F29A	−10.6 (16)
C34—P1—O6—C7	−85.9 (7)	C19—C23—F30—F29A	105.7 (17)
Pd1—P1—O6—C7	32.4 (7)	F30A—F28—F28A—C23	25 (2)
P1—O6—C7—C10	155.1 (6)	C23—F28—F28A—F29	−19 (2)
P1—O6—C7—C11	−88.7 (8)	F30A—F28—F28A—F29	6(4)
P1—O6—C7—C8	32.9 (10)	F29A—C23—F28A—F28	−136 (3)
O6—C7—C8—N9	−70.3 (10)	F30A—C23—F28A—F28	−23 (2)
C10—C7—C8—N9	174.0 (8)	F30—C23—F28A—F28	−69 (3)
C11—C7—C8—N9	51.8 (10)	F29—C23—F28A—F28	−160 (2)
O6—C7—C8—C15	116.6 (9)	C19—C23—F28A—F28	96 (2)
C10—C7—C8—C15	0.9 (13)	F29A—C23—F28A—F29	24.7 (16)
C11—C7—C8—C15	−121.2 (10)	F30A—C23—F28A—F29	136.9 (16)
C15—C8—N9—C12	3.8 (13)	F28—C23—F28A—F29	160 (2)
C7—C8—N9—C12	−169.4 (8)	F30—C23—F28A—F29	91 (2)
C15—C8—N9—Pd1	−158.1 (7)	C19—C23—F28A—F29	−103.5 (13)
C7—C8—N9—Pd1	28.7 (10)	F29A—F29—F28A—F28	−10 (3)
C1—Pd1—N9—C12	−64 (6)	C23—F29—F28A—F28	22 (2)
N5—Pd1—N9—C12	52.0 (7)	F29A—F29—F28A—C23	−31.9 (19)
P1—Pd1—N9—C12	−133.9 (7)	F28A—F29—F29A—C23	28.3 (15)
C1—Pd1—N9—C8	98 (6)	C23—F29—F29A—F30	−20 (3)
N5—Pd1—N9—C8	−145.8 (7)	F28A—F29—F29A—F30	8(4)
P1—Pd1—N9—C8	28.3 (7)	F30A—C23—F29A—F29	−146.0 (19)
C8—N9—C12—C13	−3.9 (14)	F28—C23—F29A—F29	−71 (3)
Pd1—N9—C12—C13	158.9 (8)	F30—C23—F29A—F29	−165 (2)
N9—C12—C13—C14	0.4 (15)	F28A—C23—F29A—F29	−37 (2)
C12—C13—C14—C15	3.0 (15)	C19—C23—F29A—F29	92 (2)
N9—C8—C15—C14	−0.4 (14)	F30A—C23—F29A—F30	18.6 (17)
C7—C8—C15—C14	172.3 (9)	F28—C23—F29A—F30	93 (3)
C13—C14—C15—C8	−3.1 (15)	F28A—C23—F29A—F30	128.0 (16)
O6—P1—C16—C17	135.4 (8)	F29—C23—F29A—F30	165 (2)
C34—P1—C16—C17	33.8 (9)	C19—C23—F29A—F30	−103.7 (15)
Pd1—P1—C16—C17	−108.1 (8)	F30A—F30—F29A—F29	−2(5)
O6—P1—C16—C21	−40.0 (8)	C23—F30—F29A—F29	23 (3)
C34—P1—C16—C21	−141.6 (7)	F30A—F30—F29A—C23	−24 (2)
Pd1—P1—C16—C21	76.5 (8)	F29A—F30—F30A—C23	22.2 (19)
C21—C16—C17—C18	5.7 (14)	F29A—F30—F30A—F28	−5(3)
P1—C16—C17—C18	−169.9 (8)	C23—F30—F30A—F28	−27.4 (15)
C21—C16—C17—C22	−177.1 (9)	F29A—C23—F30A—F30	−21 (2)
P1—C16—C17—C22	7.3 (14)	F28—C23—F30A—F30	−150.0 (19)
C16—C17—C18—C19	−1.2 (16)	F28A—C23—F30A—F30	−134.9 (17)
C22—C17—C18—C19	−178.5 (9)	F29—C23—F30A—F30	−56 (3)
C17—C18—C19—C20	−3.7 (15)	C19—C23—F30A—F30	103.0 (16)
C17—C18—C19—C23	177.1 (10)	F29A—C23—F30A—F28	128.6 (19)
C18—C19—C20—C21	3.7 (16)	F30—C23—F30A—F28	150.0 (19)
C23—C19—C20—C21	−177.1 (10)	F28A—C23—F30A—F28	15.1 (16)
C19—C20—C21—C16	1.2 (16)	F29—C23—F30A—F28	94 (3)
C19—C20—C21—C24	−177.7 (10)	C19—C23—F30A—F28	−106.9 (15)
C17—C16—C21—C20	−5.7 (14)	F28A—F28—F30A—F30	2(4)
P1—C16—C21—C20	170.1 (8)	C23—F28—F30A—F30	30.5 (17)
C17—C16—C21—C24	173.0 (9)	F28A—F28—F30A—C23	−29 (3)
P1—C16—C21—C24	−11.2 (13)	O6—P1—C34—C35	127.6 (8)
C18—C17—C22—F26	−148.1 (9)	C16—P1—C34—C35	−129.6 (8)
C16—C17—C22—F26	34.6 (13)	Pd1—P1—C34—C35	15.9 (8)
C18—C17—C22—F27	−28.5 (13)	O6—P1—C34—C39	−30.7 (7)
C16—C17—C22—F27	154.2 (9)	C16—P1—C34—C39	72.0 (8)
C18—C17—C22—F25	91.1 (10)	Pd1—P1—C34—C39	−142.4 (6)
C16—C17—C22—F25	−86.2 (11)	C39—C34—C35—C36	9.2 (13)
C20—C19—C23—F29A	57 (2)	P1—C34—C35—C36	−149.8 (8)
C18—C19—C23—F29A	−124 (2)	C39—C34—C35—C40	−167.8 (9)
C20—C19—C23—F30A	−63.4 (19)	P1—C34—C35—C40	33.1 (13)
C18—C19—C23—F30A	115.8 (17)	C34—C35—C36—C37	−1.8 (15)
C20—C19—C23—F28	−137.2 (16)	C40—C35—C36—C37	175.7 (9)
C18—C19—C23—F28	42 (2)	C35—C36—C37—C38	−5.7 (16)
C20—C19—C23—F30	−10 (2)	C35—C36—C37—C41	175.7 (10)
C18—C19—C23—F30	169.1 (15)	C36—C37—C38—C39	5.2 (16)
C20—C19—C23—F28A	−178.3 (15)	C41—C37—C38—C39	−176.2 (10)
C18—C19—C23—F28A	1(2)	C37—C38—C39—C34	2.7 (15)
C20—C19—C23—F29	101.9 (15)	C37—C38—C39—C42	−172.7 (9)
C18—C19—C23—F29	−78.9 (15)	C35—C34—C39—C38	−9.6 (13)
C20—C21—C24—F32	−90.2 (10)	P1—C34—C39—C38	150.0 (8)
C16—C21—C24—F32	91.0 (11)	C35—C34—C39—C42	164.9 (9)
C20—C21—C24—F33	145.1 (9)	P1—C34—C39—C42	−35.4 (13)
C16—C21—C24—F33	−33.7 (13)	C36—C35—C40—F43	177.0 (9)
C20—C21—C24—F31	29.2 (12)	C34—C35—C40—F43	−5.7 (15)
C16—C21—C24—F31	−149.6 (9)	C36—C35—C40—F44	53.4 (11)
F29A—C23—F28—F28A	62 (3)	C34—C35—C40—F44	−129.3 (10)
F30A—C23—F28—F28A	155 (3)	C36—C35—C40—F45	−63.7 (10)
F30—C23—F28—F28A	131 (2)	C34—C35—C40—F45	113.6 (10)
F29—C23—F28—F28A	20 (2)	C38—C37—C41—F46	167.1 (13)
C19—C23—F28—F28A	−101 (2)	C36—C37—C41—F46	−14.3 (18)
F29A—C23—F28—F30A	−93 (3)	C38—C37—C41—F47	−69.5 (17)
F30—C23—F28—F30A	−24.0 (16)	C36—C37—C41—F47	109.1 (14)
F28A—C23—F28—F30A	−155 (3)	C38—C37—C41—F48	47.5 (15)
F29—C23—F28—F30A	−135.3 (16)	C36—C37—C41—F48	−133.9 (13)
C19—C23—F28—F30A	104.0 (16)	C38—C39—C42—F50	−62.7 (11)
F30A—C23—F29—F29A	54 (3)	C34—C39—C42—F50	122.4 (10)
F28—C23—F29—F29A	131 (2)	C38—C39—C42—F51	173.7 (9)
F30—C23—F29—F29A	14 (2)	C34—C39—C42—F51	−1.3 (15)
F28A—C23—F29—F29A	144 (2)	C38—C39—C42—F49	55.3 (11)
C19—C23—F29—F29A	−106 (2)	C34—C39—C42—F49	−119.7 (10)

### Hydrogen-bond geometry (Å, °)

**Table:**

*D*—H···*A*	*D*—H	H···*A*	*D*···*A*	*D*—H···*A*
C4—H4B···F1^i^	0.98	2.40	3.365 (13)	166
C12—H12···F5^i^	0.95	2.60	3.276 (11)	129
C4—H4A···F4	0.98	2.53	3.464 (13)	158
C4—H4C···F2^ii^	0.98	2.30	3.225 (13)	155
C11—H11C···F5^iii^	0.98	2.49	3.352 (11)	146

Symmetry codes: (i) −*x*+1, −*y*+1, −*z*; (ii) *x*+1, *y*, *z*; (iii) −*x*, −*y*+1, −*z*.
